# Sensitivity Analysis and Accuracy of a CFD-TFM Approach to Bubbling Bed Using Pressure Drop Fluctuations

**DOI:** 10.3389/fbioe.2017.00038

**Published:** 2017-06-24

**Authors:** Leonardo Tricomi, Tommaso Melchiori, David Chiaramonti, Micaël Boulet, Jean Michel Lavoie

**Affiliations:** ^1^Department of Chemical Engineering and Biotechnology, University of Sherbrooke, Sherbrooke, QC, Canada; ^2^RE-CORD, Department of Industrial Engineering, University of Florence, Florence, Italy; ^3^Enerkem Inc., Sherbrooke, QC, Canada

**Keywords:** Eulerian–Eulerian two fluid model, fluidized bed, pressure drop oscillations, power spectral density, interphase drag law

## Abstract

Based upon the two fluid model (TFM) theory, a CFD model was implemented to investigate a cold multiphase-fluidized bubbling bed reactor. The key variable used to characterize the fluid dynamic of the experimental system, and compare it to model predictions, was the time-pressure drop induced by the bubble motion across the bed. This time signal was then processed to obtain the power spectral density (PSD) distribution of pressure fluctuations. As an important aspect of this work, the effect of the sampling time scale on the empirical power spectral density (PSD) was investigated. A time scale of 40 s was found to be a good compromise ensuring both simulation performance and numerical validation consistency. The CFD model was first numerically verified by mesh refinement process, after what it was used to investigate the sensitivity with regards to minimum fluidization velocity (as a calibration point for drag law), restitution coefficient, and solid pressure term while assessing his accuracy in matching the empirical PSD. The 2D model provided a fair match with the empirical time-averaged pressure drop, the relating fluctuations amplitude, and the signal’s energy computed as integral of the PSD. A 3D version of the TFM was also used and it improved the match with the empirical PSD in the very first part of the frequency spectrum.

## Introduction

Fluidized bubbling reactors are extensively employed in the industry, both for chemical and biochemical processes as well as for power generation, and one of the main reasons is due to their optimal level of heat and mass transfer induced by the bubbling turbulence (Singh et al., 2013). Under this regime, bubbles are responsible for the overall mixing among phases, and it is hence essential to understand their fluid dynamics to optimize the whole process. Improving reactors efficiency while at the same time reducing their CAPEX and OPEX is still a source of numerous investigations in literature (Singh et al., 2013). Today, small- to medium-scale fluidized bed and their applications are studied using CFD models throughout different numerical approaches offering different types of accuracy (as well as different computational costs). These latter represent a very important barrier when modeling complex systems such as bubbling fluidized beds (BFBs), and research is actively focusing on reducing the computational requirement of numerical models while improving their accuracy. In multiphase applications, where the solid phase involves a very high number of particles, the Eulerian–Eulerian two fluid model (TFM) has been proven to be the most convenient investigation approach (Singh et al., 2013). In addition to this method, two possible alternatives for describing the fluid dynamic of a multiphase granular system are the Eulerian–Lagrangian discrete particle model and the direct numerical simulation. These two methods, and especially the latter, are well known for their accuracy in estimating the particles trajectories while providing a full detailed map of the fluid patterns inside the system. However, their application to dense particle systems is not an easy task since they require massive computational cost especially when describing a large amount of particles (in addition to their countless interactions). The TFM represents a convenient mathematical way to model dense particles system because of its intrinsic quicker performance (when compared with the aforementioned approaches) in capturing and providing information about bubble shapes, motions as well as on the bed expansion.

Among the different experimental strategies that could be used to study and monitor the bubbling process, pressure fluctuation is one of the most convenient ones since it is easy to measure and can be directly linked to the bubbles dynamics. Numerous studies have investigated the coupling between bubbles dynamics and pressure fluctuations as a convenient way to characterize the transient behavior of a bubbling multiphase system, from the early works published by Davidson and Harrison (1963) up to more recent studies (Johnsson and Johnsson, 2001; Peirano et al., 2001; Acosta-Iborra et al., 2011).

Despite these advantages, the interpretation of pressure fluctuations is both complicated and challenging since there are various sources involved in generating this signal (Bi, 2007). Qingcheng et al. (2011) observed the physical phenomenon of a bubble formation and motion rising up through the solid particles bed and found in this process the main source of perturbation of the gas–solid system. While linking the local pressure fluctuations to the bubbles presence and movement, they also assessed the influence of the operating gas velocity on the overall amplitude of pressure drop as well as on their major frequency.

Peirano et al. (2001) conducted a CFD study of a BFB using an Eulerian TFM approach. In their study, they highlighted the importance of pressure drop low frequencies because of their direct connection with the bubbling motion. Nevertheless a clear interpretation about the origin of the higher frequencies was not provided. Furthermore, they assessed the suitability of a 2D model as far as the sensitivity analysis is regarded while recommending a full 3D modeling when attempting to catch the dynamic of the real system. A similar conclusion was also found by Acosta-Iborra et al. (2011) who performed differential pressure spectrum analysis along with particle fraction spectrum. While showing the close relation of these two spectrums and consequently the local character of the information provided by differential pressure probes, they also advised the use of a full 3D simulation to catch the bubble coalescence and interaction with the surface of the bed.

The primary importance of the fluid-particle drag, as the main driving force in cold fluid dynamic systems, is often noticed in open literature and represents one of the key points to achieve a good prediction of bubbling bed hydrodynamic. In general, the drag law depends on a drag coefficient (*C_D_*), which in its turn depends on the local relative velocity between phases and the void fraction. This coefficient depends as well on other factors such as particle size distribution and particle shape. However, it is difficult to characterize the void fraction dependency for any conditions other than for a packed bed or for infinite dilution (single particle model, Vejahati et al., 2009). To bypass this lack of crucial data, some authors attempted to exploit the experimental minimum fluidization velocity of their own system as a calibration point. For example, Syamlal and O’Brien (1987) introduced a method to adjust the drag law using the *U*_mf_ value of their system (Syamlal and O’Brien, 1988). This approach allows calibrating (before starting the simulations) a special correlation between a single and a multiple particle systems under settling condition. Esmaili and Mahinpey (2011) compared the results of their 3D TFM to empirical data using time-averaged pressure drop at different locations as well as bed expansion ratio. They specifically focused on the effect brought by different drag formulations, finding the parametric Syamlal–O’Brien drag law (Syamlal and O’Brien, 1987) as one of the best for providing a correct prediction of these two indicators over the wide range of superficial velocities investigated. Min et al. (2010) validated their 2D and 3D TFM throughout gas holdup measurements (using X-ray imaging system) as well as by the time-averaged pressure drop data. They also focused on the effect brought by different formulations of the drag law. Both their 2D and 3D model correctly predicted the experimental time-averaged pressure drop and also, in this case, the Syamlal–O’Brien drag formulation showed a better prediction of the gas holdup variation through the bed height.

This drag law was used in this work because of its intrinsic superior capability to provide the best prediction for solid bed expansions, bubbling displacement and foremost, by matching the experimental pressure drop.

While it is clear that model validation cannot be achieved by means of mere time-averaged pressure drop (since no information related to the bed dynamic can be recovered out of it) the stochastic behavior of bubbles do not allow having an univocal time signal that could be used as a validation point. However, these limitations can be rounded up by performing spectrum analysis to obtain a frequency distribution, which is univocal of any specific operating condition setup (bed height, air velocity, particle size, etc.). Even though a few studies went through the analysis of pressure fluctuations (by performing spectrum analysis), information about the (sampling) time scale required to fully catch the “finger prints” of pressure drop fluctuations through the bed has not being investigated in depth.

One major target of this work is to test the effect of sampling time on the empirical pressure drop oscillations spectra (PSD) to limit the duration of CFD simulations while ensuring the validation of CFD model with empirical data. This work will investigate the numerical sensitivity of a TFM model applied to a BFB reactor, to better understand the impact of certain parameters on the accuracy that such model can provide once compared to the empirical data. To this purpose the model was tested on a 2D geometry employing the parametric Syamlal–O’Brien drag law. For each parameter, a specific set of simulations have been performed by varying its value or the related mathematical formulation. The results have been compared in terms of time-averaged pressure drop, variance, and signal energy. Numerical verification was also carried out, prior to the model sensitivity analysis, identifying the maximum mesh size and therefore guaranteeing the convergence of the numerical solution. A full 3D model was also implemented and used to improve the numerical accuracy, ultimately resulting in a better fit with the first part of the empirical PSD.

## Experimental Setup

**Figure 1:**
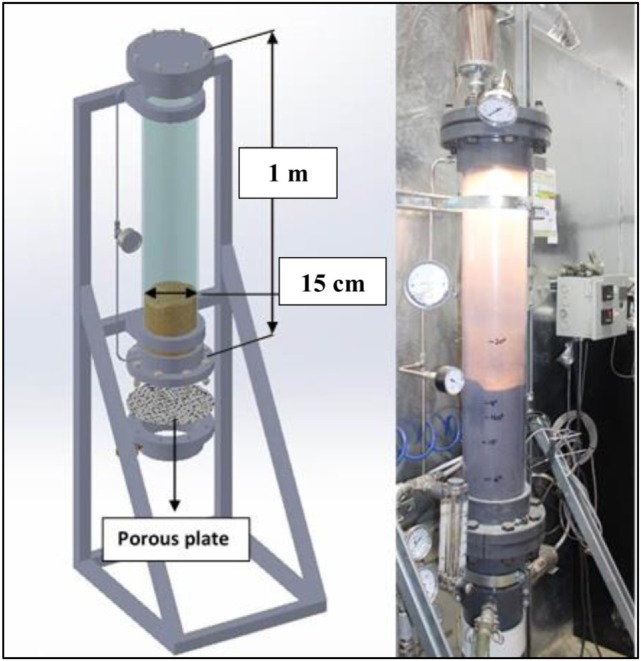
Schematic of test apparatus (left) and real laboratory scale bench (right) used for the first part of this work.

The experimental setup used in this work (shown in Figure 1) has been chosen following the assembling method discussed in ASTM International (2012). The latter comprises of a lab-scale fluidized bed and specific instrumentation measuring and monitoring both the gas flow discharge and the pressure drop along the bed. In the actual work the reactor body is made of clear PVC, which has been selected to allow a dynamic visual analysis of the process. The body of this system is a 15 cm i.d. over a 1 m height cylinder. The bottom flange allows stabilization of the base of the PVC cylinder wall while embedding the porous gas distributor plate. This latter is stainless 316 L made and presents a microporosity of 1.3 μm such as to ensure an optimal homogenization of the gas prior to the reactor inlet. The choice of such a distributor typology is dual, first contributing to generate small bubbles all over the cross section while ultimately helping avoiding some experimental drawbacks like dead spaces and the back sitting of solids. Second, it allows an easier numerical schematization of the inlet boundary condition that can be accounted easily into a 2D geometry, differently from what it would be required by other types of air injectors (such as nozzles) where the 3D model would be the only possible choice. This last aspect is crucial to perform CFD simulations with significant time economy in the early stages of model implementation and verification. Moreover, the very fine porosity is such to guarantee a local pressure drop (induced by its own intrinsic porosity) comparable to the one along the bed in the fluidization regime. Despite being highly conservative, this precaution is always considered when designing a proper gas distributor to avoid a potential and persistent gas channeling inside the bed induced by a too low pressure drop. However, at industrial scale, porous plates are not often employed hence avoiding the risks of clogging which could be induced by inert material (that does not fluidize) as well as other compounds that might melt on the distributor surface.

A filter is placed on top of the upper flange to prevent solid particles from being entrained out of the bed during fluidization regime and, right next to it, a relief valve allowing to avoid any dangerous overpressures. For the tests, the reactor was operated under ambient conditions. The key device, for validation purposes, was a differential pressure gage (Kistler 4264A), capable of recording up to 1,000 pressure drop data per second. These latter were then transferred to a Labview acquisition system for data saving and real time-pressure drop monitoring. The pressure drop was measured between two points at the extremities of the cylinder’s body. The bottom probe was positioned at 2.5″ over the porous plate, and the upper one was at the proximity of the top flange. Two small meshed screens were put inside the two pipes of the differential pressure gage to avoid particles entrainment and therefore potential damages to the instrument. Two flow meters were included in the setup, one manual (rotameter) potentially available to measure high air flows, and the other was an electronic unit operating in the range 0–300 SLPM. Experiments were performed at 22°C (room temperature) and 1 atm, conditions that remained constant during the tests. Finally a small light bulb was located in the upper interior section of the reactor flange, lighting up the bed surface hence allowing to take better quality pictures and videos.

The bed material used is alumina powder (190 μm Sauter diameter) belonging to the Geldart Group B. Alumina was selected since it is often used in industrial-scale gasifiers (where this inert represents by far the major part of the total solid bed mass). The particular size allowed covering a good range of hydrodynamic conditions (from fixed bed to vigorous bubbling condition) since the minimum fluidization velocity is strongly linked to the diameter of solid particle. By doing so, the system could be operated without the need for a manual flowmeter, whose reading accuracy, could be considerably lower than the electronic unit. Gas and solid properties used for both experiments and corresponding CFD simulations are listed in Table 1.

**Table 1:** Materials physical properties for the experimental gas–solid system.

Material	Properties	Units	Value
Alumina	Particle diameter	μm	190
	Particle density	kg m^−3^	3,883
	Particle sphericity	–	0.6
	Coefficient of restitution	–	0.85
	Static bed height	mm	263
	Packing limit	–	0.54
	Friction packing limit	–	0.48
	Initial solid volume fraction	–	0.52
	Angle of internal friction	–	60°
Air	Density	kg m^−3^	2.417
	Viscosity	N s m^−2^	1.8 × 10^−5^

The bench reactor was filled with alumina up to a bed height of 263 mm, corresponding to a total mass of approximately 9.5 kg. Different superficial velocity values below the minimum fluidization one were exploited for the CFD validation in the fixed regime, whereas only one value corresponding to 3.5 times the minimum fluidization velocity was used for validating the CFD model in the “bubbling” regime. This value was selected to guarantee a vigorous fluidization regime while respecting a margin of accuracy for the electronic air flow reading.

## Hydrodynamic and Numerical Model

This cold system includes gas and solid particles mixed together in an enclosed cylindrical vessel where the bubbles are generated at the very bottom of the reactor when the superficial velocity of the gasifying agents exceeds the minimum fluidization value. In this work, CFD analysis is meant to predict the effect of bubble formation as well as their motion toward the bed surface. It should also allow predicting the pressure drop oscillations induced by bubble patterns and chaotic particles displacement. The model considers both the gas (generally air in cold fluid dynamic applications) and the solid phase as two interpenetrating fluids for which conservation equations (mass and momentum) are derived. However, these equations require a proper closure, which can be provided by the constitutive/rheological laws. The latter are obtained from empirical correlations and by application of the kinetic theory of granular flows (KTGFs). The general form of the TFM equations is the following.

Continuity equation (valid for both gas and solid phase)

(1) $$\frac{\partial }{\partial t}\alpha_{q}\rho_{q}+\nabla \cdot \alpha_{q}\rho_{q}{\vec{u}}_{q}=\sum_{p=1}^{n}{ṁ}_{\mathrm{pq}}$$

where α*_q_* is the volume fraction of phase *q* (here representing either the gas or the solid phase), ρ*_q_* its density, and ${\vec{u}}_{q}$ the corresponding velocity vector. The term ${ṁ}_{\mathrm{pq}}$ represents the mass transfer between phases (kg m^−3^ s^−1^). By definition, the sum of the phase fractions α*_q_* is equal to 1.

Gas phase momentum equation

(2) $$\frac{\partial }{\partial t}\alpha_{g}\rho_{g}{\vec{u}}_{g}+\nabla \cdot \left(\alpha_{g}\rho_{g}{\vec{u}}_{g}⊗{\vec{u}}_{g}\right)=-\alpha_{g}\nabla P+\nabla \cdot \alpha_{g}{\bar{\bar{\tau }}}_{g}+\alpha_{g}\rho_{g}\vec{g}+K_{\mathrm{gs}}\left({\vec{u}}_{s}-{\vec{u}}_{g}\right)$$

where *P* represents the operating pressure inside the system, *g* the gravity, and *K_gs_* the drag factor of phase *s* in phase *g* (kg m^−3^ s^−1^).

The gas stress tensor is given by:

(3) $${\bar{\bar{\tau }}}_{g}=\mu_{g}\left(\nabla {\vec{u}}_{g}+{\left(\nabla {\vec{u}}_{g}\right)}^{T}\right)+\left(\lambda_{g}-\frac{2}{3}\mu_{g}\right)\nabla {\vec{u}}_{g}\cdot I$$

Solid phase momentum equation

(4) $$\frac{\partial }{\partial t}\alpha_{s}\rho_{s}{\vec{u}}_{s}+\nabla \cdot \left(\alpha_{s}\rho_{{\;}_{s}}{\vec{u}}_{s}⊗{\vec{u}}_{s}\right)=-\alpha_{s}\nabla P-\nabla P_{s}+\nabla \cdot \alpha_{s}{\bar{\bar{\tau }}}_{s}+\alpha_{s}\rho_{s}g+K_{\mathrm{gs}}\left({\vec{u}}_{g}-{\vec{u}}_{s}\right)$$

where α*_s_* is the volume fraction of the solid phase s and ${\vec{u}}_{s}$ (m s^−1^) is the corresponding velocity vector. All the other terms are explained in the following.

Also for the solid phase, the total viscous stress tensor is expressed by the following expression:

(5) $${\bar{\bar{\tau }}}_{s}=\mu_{s,\text{tot}}\left(\nabla {\vec{u}}_{s}+{\left(\nabla {\vec{u}}_{s}\right)}^{T}\right)+\left(\lambda_{s}-\frac{2}{3}\mu_{s}\right)\nabla {\vec{u}}_{s}\cdot I$$

where the viscosity coefficients include the combination of different terms:

(6) $$\mu_{s,\text{tot}}=\mu_{s,\text{col}}+\mu_{s,\text{kin}}+\mu_{s,\text{frict}}$$

where μ_*s*,tot_ is the total solid shear viscosity resulting from the summation of three different components, which are described below and are correspondingly the collisional (Gidaspow et al., 1992; Syamlal et al., 1993), kinetic (Gidaspow et al., 1992), and frictional (Schaeffer, 1987) components of the total sheer stress.

(7) $$\mu_{s,\text{col}}=\frac{4}{5}\alpha_{s}\rho_{s}d_{s}g_{o,\mathrm{ss}}\left(1+e_{\mathrm{ss}}\right){\left(\frac{\Theta_{s}}{\pi }\right)\;\;}^{1∕2}\alpha_{s}$$

(8) $$\mu_{s,\text{kin}}=\frac{10\alpha_{s}\rho_{s}d_{s}\sqrt{\Theta_{s}\pi }}{96\alpha_{s}\left(1+e_{\mathrm{ss}}\right)g_{o,\mathrm{ss}}}{\left[1+\frac{4}{5}g_{o,\mathrm{ss}}\alpha_{s}\left(1+e_{\mathrm{ss}}\right)\right]\;}^{2}$$

(9) $$\mu_{s,\text{frict}}=\frac{P_{s}\;\text{sin}\Phi }{2\sqrt{I_{2D}}}$$

where α*_s_* represents the solid volume fraction, Θ*_s_* (m^2^ s^−2^) the granular temperature, *g_o,ss_* the radial distribution (Ogawa et al., 1980), *d_s_* (m) the solid particle diameter (Sauter), and *P_s_* the total solid pressure [below the expression from Lun et al. (1984)]. *P*_frict_ is a frictional component (Johnson and Jackson, 1987), λ*_s_* is the solid bulk viscosity (Lun et al., 1984) accounting for the resistance of the granular flow to compression and expansion, and *e_ss_* is the restitution coefficient expressing the ratio between the particle speed after and before collisions. Mathematical description of these variables is given by the following:

(10) $$\Theta_{s}=\frac{1}{3}\;\left\langle {\vec{u}}_{s}^{\prime }\cdot {\vec{u}}_{s}^{\prime }\right\rangle$$

(11) $$g_{o,\mathrm{ss}}={\left[1-{\left(\frac{\alpha_{s}}{\alpha_{s,\text{max}}}\right)}^{\;1∕3}\right]}^{\;-1}$$

(12) $$\lambda_{s}=\frac{4}{3}\alpha_{s}\rho_{s}d_{s}g_{s}\left(1+e_{\mathrm{ss}}\right){\left(\frac{\Theta_{s}}{\pi }\right)}^{\;\;1∕2}\alpha_{s}$$

(13) $$P_{\text{frict}}=\mathrm{Fr}\frac{{\left(\alpha_{s}-\alpha_{s,\text{min}}\right)}^{2}}{{\left(\alpha_{s}-\alpha_{s,\text{max}}\right)}^{5}}$$

(14) $$P_{s}=\alpha_{s}\rho_{s}\Theta_{s}+2\rho_{s}\left(1+e_{\mathrm{ss}}\right){\alpha_{s}}^{2}g_{o,\mathrm{ss}}\Theta_{s}$$

(15) $$P_{s}=2\rho_{s}\left(1+e_{\mathrm{ss}}\right){\alpha_{s}}^{2}g_{o,\mathrm{ss}}\Theta_{s}$$

(16) $$P_{s}=\alpha_{s}\rho_{s}\Theta_{s}\left[\left(1+4\alpha_{s}g_{o,\mathrm{ss}}\right)+\frac{1}{2}\left[\left(1+e_{\mathrm{ss}}\right)\left(1-e_{\mathrm{ss}}+2\mu_{\text{fric}}\right)\right]\right]$$

### Drag Law Formulation

The last term on the RHS both for Eqs 2 and 4 represents the drag force causing the interphase momentum exchange between the gas and solid phases. This term is by far the predominant one in cold systems, and its formulation can significantly affect the CFD outputs (Esmaili and Mahinpey, 2011).

The drag force depends in general of the local relative velocity between phases and the void fraction but also on some other factors such as the particle size distribution and particle shape. The particle void fraction is, however, very difficult to be determined other than in a packed bed or infinite dilution (single particle). Other factors such as particle shape, clustering, and particle size distribution can also affect the local drag force, but they have never been considered in deriving drag correlations (Vejahati et al., 2009). Syamlal and O’Brien (1987) derived a formula for the fluid–solid drag coefficient for multiparticle system using the Richardson–Zaki type velocity–voidage correlation (Richardson and Zaki, 1954). Based on the terminal velocity of particles in fluidized or settling beds, the authors proposed the following drag correlation:

(17) $$K_{\mathrm{gs}}=\frac{3}{4}\frac{C_{D}}{v_{r,s}^{2}}\frac{\rho_{g}\left|{\vec{u}}_{s}-{\vec{u}}_{g}\right|}{d_{s}}\alpha_{g}\alpha_{s}$$

where

(18) $$C_{D}={\left(0.63+\frac{4.8}{\sqrt{{\text{Re}}_{s}∕v_{r,s}\;}}\right)}^{\;2}$$

(19) $${\text{Re}}_{s}=\frac{\rho_{g}d_{s}\left|{\vec{u}}_{s}-{\vec{u}}_{g}\right|}{\mu_{g}}$$

(20) $$v_{r,s}=0.5\;\;\left[A\;-\;0.06\text{Re}+\sqrt{0.0036{\text{Re}}^{2}\;+\;0.12\text{Re}\left(2B\;-\;A\right)+A^{2}}\right]$$

with

(21) $$A=\alpha_{g}^{4.14}$$

(22) $$B=\left\{\begin{array}{ll} \alpha_{g}^{C_{1}}\; & \text{if}\;\alpha_{g}\geq \;0.85\\ C_{2}\alpha_{g}^{1.28}\; & \text{if}\;\alpha_{g}<\;0.85\\ C_{1}\;=\;2.65\; & \text{and}\;C_{2}\;=\;0.8 \end{array}\right.$$

However, the Syamlal–O’Brien drag model presented above (with constant coefficients *C*_1_ and *C*_2_) can result in the under/over prediction of the minimum fluidization velocity and consequently in a too high/low bed expansion (ANSYS, 2012). To cope with this drawback, a parametric version of the Syamlal–O’Brien drag model was used in this work. This parametric drag model exploits the minimum fluidization velocity and void fraction (on the fluidization onset) as a calibration point to adjust the drag force. Both these two parameters have to be experimentally measured and provided to the (drag model) inner algorithm which performs an iteration process to minimize the following objective function:

(23) $$\left\{U_{\mathrm{mf}}^{\exp\;\mathrm{eriment}}-{\text{Re}}_{t}\frac{\alpha_{g}\mu_{g}}{d_{s}\rho_{g}}\right\}\overset{\mathrm{Min}}{\to }\;0$$

where

(24) $${\text{Re}}_{t}=v_{r,s}{\text{Re}}_{\mathrm{ts}}$$

(25) $$v_{r,s}=\frac{A+0.06B{\text{Re}}_{\mathrm{ts}}}{1+0.06{\text{Re}}_{\mathrm{ts}}}$$

(26) $${\text{Re}}_{\mathrm{ts}}={\left(\frac{\sqrt{4.8^{2}+2.52\sqrt{4\text{Ar}∕3\;}}-4.8}{1.26}\right)}^{2}$$

(27) $$\mathrm{Ar}=\frac{\left(\rho_{s}-\rho_{g}\right)d_{s}^{3}\rho_{g}g}{\mu_{g}^{2}}$$

with

(28) $$A=\alpha_{g}^{4.14}$$

(29) $$B=\left\{\begin{array}{ll} \alpha_{g}^{d_{1}}\; & \text{if}\;\alpha_{g}\geq \;0.85\\ C_{2}\alpha_{g}^{1.28}\; & \text{if}\;\alpha_{g}<\;0.85\\ d_{\text{1}}=1.28+\frac{\log\left(C_{\text{2}}\right)}{\log\left(0.85\right)} \end{array}\right.$$

(30) $$C_{D}\left(\text{Re},\alpha_{g}\right)={\left(0.63+\frac{4.8}{\sqrt{\text{Re}∕v_{r,s}\;}}\right)}^{2}$$

where Re*_t_* represents the Reynold number of a multiparticle system at the fluidization onset (minimum fluidization velocity or settling condition), Re*_ts_* corresponding number for one single particle, *Ar* the Archimede number, *C_D_*(Re,α*_g_*) an analytical expression for the multiparticle drag coefficient, and *v_r_* is the terminal velocity for the solid phase as derived by the velocity–voidage correlation proposed by Garside and Al-Dibouni (1977). According to an algorithm, the parameter *C*_2_ (and consequently *d*_1_) is changed until the objective function (relation 23) is minimized. Hence, a new set of two parameters is obtained, giving a more accurate estimation of the drag coefficient for any dynamic condition inside the bed (Re and α*_g_*) as shown by Eq. 30. The main critical point of this parametric drag law is given by the necessity to provide very precise values both for the minimum fluidization velocity and the air (void) volume fraction (since the CFD model is really sensitive to both). Thus, these couple of values are to be provided to the CFD model according to the estimated experimental values on the onset of fluidization. However, especially regarding the determination of the *U*_mf_, there is always a margin of uncertainty since from experiments, there is a not a clear limit of gas velocity marking the transition from fix regime to bubbling. To cope with this uncertainty, a series of simulations (as reported in Sections “TFM vs Experiments: Model Validation Methodology” and “Model Sensitivity Analysis”) were performed using different minimum fluidization velocities. The second parameter (bed void fraction) was determined univocally and according to the bed’s weight and corresponding volume occupied inside the bed at the fluidization onset.

As shown above (Eqs 14–16), in this work three different formulation for the solid pressure term (*P_s_*) have been considered to test the model sensitivity analysis with regards to this parameter as discussed in Section “Model Sensitivity Analysis.” Based upon the KTGF, an algebraic formulation (obtained neglecting the convection and diffusion term) of the conservation of energy for the solid particles was used to work as a closure for the solid stress tensor (Eq. 5).

## Numerical Simulation

Numerical simulations were performed using Ansys-Fluent 16.2 and ran on high performance computing (HPC) at the University the Sherbrooke (*Mammoth Parallel II*). The software adopted proper numerical methods for discretizing and solving the set of equations shown in Section “Hydrodynamic and Numerical Model.” The Eulerian–Eulerian TFM approach accounts for a set of conservation equations for each phase.

Based upon The Finite Volume approach, as the general framework for discretizing and integrating main equations, a Phase-Coupled Semi Implicit Method for Pressure Linked Equations was used, thus extending the SIMPLE approach to multiphase cases. According to this method, the pressure values are computed for each time step in the cell centers while the velocities components are calculated at each cell interface. In this staggered scheme, velocities and pressure are first calculated and secondly corrected according to an iterative process to respect the continuity constraint. Because of the transient formulation of the problem, an implicit second order scheme has been adopted for temporal discretization of time-derivate variables. A fixed time step of 10^−4^ s was chosen for all the simulations to ensure their stable convergence. The convergence criteria is based on the residual values of the solution (for each of the unknown variables) solved inside the numerical domain. The tolerances on residuals were set to 10^−3^ for continuity and 10^−4^ for the velocity components. For spatial discretization, the MUSCL method has been chosen to minimize numerical diffusion. In fact, as shown by Tagliaferri et al. (2013), in the full fluidization regime, the First Order Upwind (FUS) scheme (provided inside the software as default option for spatial discretization) introduces a high numerical diffusion leading to the potential risk of smoothing out the solid volume fraction gradients at bubbles boundaries and ultimately failing to predict the correct bubble size and distribution.

### Mesh Grid Sensitivity Analysis (2D Model)

**Figure 2:**
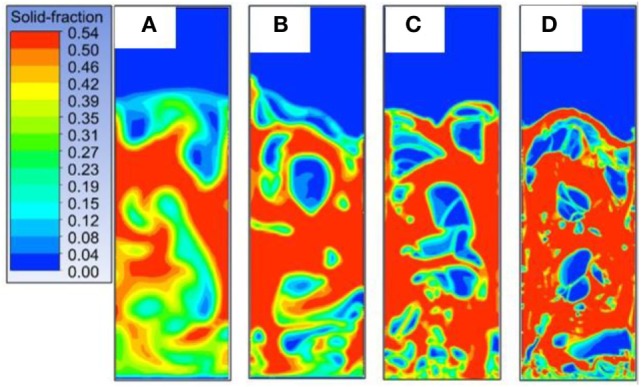
Solid volume fraction contours at time 20 s for *U* = 0.2 m s^−1^. From left to right four decreasing mesh size 7.62 **(A)**, 3.81 **(B)**, 1.905 **(C)**, and 0.635 **(D)** mm.

Based upon the numerical setup described in the previous section, a mesh grid sensitivity analysis was carried out to evaluate the convergence of numerical solutions. The performance of the CFD models (time required by the simulations to perform) are heavily affected by the choice of the mesh size. To this purpose, four simulations were carried out based on identical operating conditions (*U*_gas_ = 0.2 m s^−1^) and material properties setup (Table 1) using four different square mesh sizes. The choice of the exact mesh size was made to obtain a precise discretization of the geometry thus avoiding any cut cells within the grid. For the 3D model, only one mesh was investigated corresponding to 20 times the particles diameter. Related results and simulation performances are reported in terms of mathematical indicators in Tables 2 and 3, respectively. The solid fraction distributions in Figure 2 show the different accuracy of CFD models in displaying the bubbles shape and distribution. According to Vejahati et al. (2009), the convergence of the numerical solution could be evaluated based upon macroscopic key indicators of the bubbling bed behavior such as the time-averaged pressure drop (measured across the bed between two fixed points) and void fraction (computed as a surface time-averaged integral for a certain bed height, i.e., 8 cm in this study). Finally, the variance of the pressure drop signal was compared, and results were time averaged in the 2–40 s range thus excluding the initial unsteady state behavior of the system (see Table 2). The observation of the pressure drop and void fraction values (both time averaged) along with the contours of solid fraction led to choose grid c (1.905 mm) as the one ensuring the convergence of the overall hydrodynamic behavior. This result supports what was previously reported by van der Hoef et al. (2006), Syamlal and O’Brien (2003), and Zimmermann and Taghipour (2005), confirming the necessity to employ a mesh size less than or equal to 10 times the Sauter diameter of particles (0.19 mm) for solution grid independency.

**Table 2:** Mesh sensitivity outputs used to assess the convergence of numerical solution.

Mesh spacing (mm)	*Δ*P (kPa)	Time-averaged void fraction
Δ = 7.62 (a)	4.144	0.64
Δ = 3.81 (b)	4.119	0.59
Δ = 1.905 (c)	4.026	0.61
Δ = 0.635 (d)	4.045	0.62

**Table 3:** Simulations performances: effect of mesh refinement on the total CFD simulation time for the 2D and 3D model.

*Δ*time 2–40 s	HPC	Number of cells	Total simulation time (h)
2D—7.62 mm	16	1,333	46
2D—3.81 mm	16	5,333	63
2D—1.905 mm	16	21,333	96
2D—0.635 mm	32	19,200	264
3D—3.81 mm	48	285,000	336

*Simulations run on HPC machines (Mammoth Parallel II) at the University of Sherbrooke*.

Based on these results square meshes of 1.905 mm side, corresponding approximately to 10 times the particles Sauter diameter, were chosen to investigate the 2D model sensitivity.

## Results and Discussion

CFD simulation results were analyzed to test the TFM model sensitivity as well as its accuracy in matching empirical data. The key parameter used to assess the CFD models accuracy was the experimental pressure drop across the bed. Specifically the power spectral density (PSD) analysis was used, attempting to quantify the effect of bubbles motions and bed mass oscillation on the pressure drop signal. Once the time-dependent pressure drop signal was obtained, two other main mathematical steps were followed to investigate the pressure fluctuation distribution. First, a power spectral density (PSD) of the signal was calculated, showing the frequency distributions of these oscillations. To this purpose a fast Fourier transform (FFT) was applied to the original signal, cutting the first 2 s of each simulation to exclude the transitory behavior of the system. Then an integral calculation of this PSD distribution was computed to show the cumulate frequency growth. This last step has been put forward just to ease the reading and the interpretation of the PSD distribution itself. Moreover, it can be noticed that the final value of the PSD integral also represents the total “energy” reached by the original signal in time. Besides being an useful indicator of the bubbling vigor, this value was also used in certain case to normalize the PSD curves (dividing their cumulative distribution by this value) and make these independent from the time scale of the pressure drop signal (see Experimental Tests to Evaluate the Dependency of PSD Distribution on Time). Accordingly only the shape of the PSD growth could be observed and analyzed. To carry out the model validation, a proper campaign of measurement was carried out covering both the “fixed” and the “bubbling” bed regimes. A dedicated experimental test allowed identifying a minimum time threshold to ensure a representative PSD of the model, which will be explained in the following section.

### Experimental Tests to Evaluate the Dependency of PSD Distribution on Time

**Figure 3:**
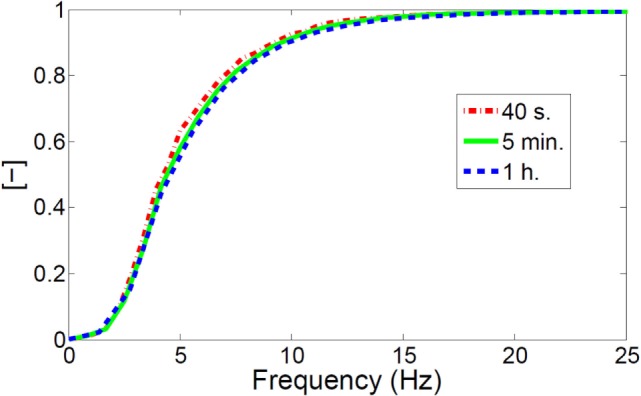
Normalized PSD integral for three different empirical tests performed in the bubbling regime (according to operating conditions reported in Section “Experimental Setup”): 40 s (red), 5 min (green), and 1 h (blue).

The time-pressure drop signal shows random pressure fluctuations because of the intrinsic stochastic behavior of bubbles. Therefore, the results are always different for a given set of geometries and operating conditions. Because of this variability, an alternative strategy would logically be required to univocally trace the “fingerprint” of bubble formation and motion inside the reactor. To this purpose, the signal was processed using FFT algorithm to obtain a frequency spectrum distribution and its corresponding integral (over the frequency domain) which, at this point, were no longer specific of the singular experiment. However, to gain a good PSD resolution, the time horizon of these experiments had to be considerably wider as compared to the one required by the single bed oscillation. Such an issue could be comparable to the choice of a representative sample size in statistics and therefore three experiments involving different duration (40 s, 5 min, 1 h) were carried out. The three corresponding normalized cumulative PSDs are plotted in Figure 3. The integral of PSD function was preferred to have a better definition of the curves. Normalization is required here to overcome the intrinsic effect of different time duration on the total energy of the original signal (which is intrinsically linked to it by definition).

All three tests show a very similar trend where the curves corresponding to tests 2 and 3 are almost overlapping while test 1 (40 s test) present minor differences due to some missing peaks in the spectrum, which ultimately results in a less regular growth of the cumulative distribution. Nevertheless, according to these results, it has been concluded that 40 s can reasonably be accepted as an end-time reference for CFD simulations. The postprocessing data of shorter tests (not reported in this work) revealed a very poor PSD distribution because of a significant lack of frequencies ultimately suggesting not to reduce any further the flow time for CFD simulations. Under the chosen numerical setup, high performance computing (HPC) can solve 40 s of real time in approximately 5 days (for the 2D model) using a 0.075″ mesh grid. The 0–25 Hz range in the frequency spectrum covers almost the entire distribution of pressure fluctuations showing that the specific fingerprints of bubbles is confined in this limited range with a major concentration of peaks in the 3–5 Hz range. The lack of a single, dominant frequency (“natural” frequency of bed mass oscillation) is not surprising and can be explained by the existence of different modes of bed oscillations which alter the natural frequency of gas–solid interactions in the fluidized bed (Ommen et al., 2011). Bi (2007) reported that these different modes are to be taken into account in such a system because of their direct impact on the pressure drop spectrum of the signal. Moreover, the major concentration of peaks in the lower part of the frequency spectrum is deemed to be strongly linked to bubbles formation and eruption as also found by Peirano et al. (2001).

### TFM vs Experiments: Model Validation Methodology

#### Fixed Regime

**Figure 4:**
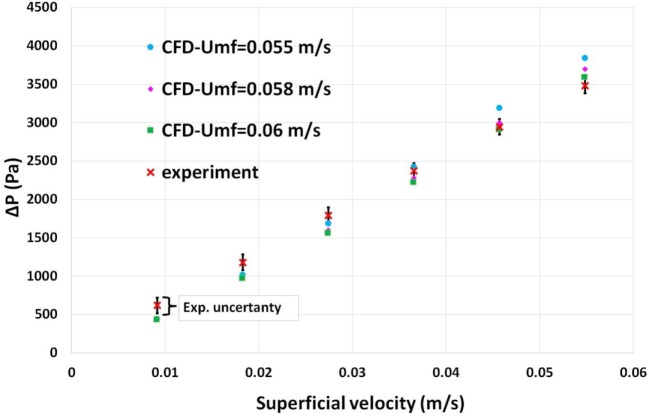
Experimental and CFDs time-averaged pressure drops for different superficial velocity tested in the fixed regime. CFD simulations were performed based on a different *U*_mf_ (used within the drag calibration algorithm). The graphic also depicts the experimental uncertainty produced by the differential pressure gage precision.

Despite the main purpose of this work being the investigation of the bubbling regime, it could be as well useful to validate the CFD model in the fixed regime. Details of the mechanical properties of the solid phase and their mathematical formulations, as implemented inside the CFD model, can be found in Table 4. The latter is valid for simulations both in the bubbling and fixed regime (with only a different definition of the frictional pressure term for fixed condition). This type of analysis was principally aimed to assess whether or not the value of minimum fluidization velocity (*U*_mf_) used inside the CFD (as one of two calibration points for our customized drag law) can also be properly predicted by the CFD model. To this purpose, six superficial velocity values were used for empirical tests and corresponding CFD simulations. As mentioned, the transition from the fixed to the bubbling regime is not abrupt, and consequently it is difficult to identify a precise and representative value of *U*_mf_. As explained in the last part of Section “Hydrodynamic and Numerical Model,” the *U*_mf_ represents, in the CFD model, an important calibration point impacting on the ultimate value of the drag coefficient. Consequently, three values of *U*_mf_ (in the range identified for the experiments) were tested by providing them as an input to the CFD model (used within the drag calibration algorithm). Three corresponding sets of simulations were performed based upon these values and the six superficial velocity used for the fixed regime as shown in Figure 4. Simulation results showed good agreement with the experimental curve where the average relative error varies around 10% for all three cases. A bigger gap was observed for lower superficial velocities and a smaller error when the bed approaches the transition to a fluidized regime. The end flow time of these simulations was set to 10 s since in the fixed regime the steady state is reached quickly. The best match with the experiments was found using a value of *U*_mf_ = 0.06 m s^−1^ (as drag law calibration point) when the superficial velocity was such as to approach the bubbling condition. Thus using the highest value of superficial velocity tested, *U_o_* = 0.0548 m s^−1^, we obtained a relative error between experiments and CFD around 1%. Results also showed that numerical results are closer to empirical values at lower superficial velocity when the smallest *U*_mf_ (0.055 m s^−1^) is used into the CFD drag law. For intermediate superficial velocities, the simulation performed using *U*_mf_ = 0.058 m s^−1^ provided better results. Consequently there is not an unique trend on the best value of *U*_mf_ to be employed into the CFD drag law and the impact of this parameter on CFD outputs was also tested for the bubbling regime.

**Table 4:** Mechanical properties of solid phase and mathematical formulation used in the CFD model (Ansys/Fluent) to simulate the gas–solid system.

Alumina (granular) properties	Units	Model
Granular temperature model	–	Phase property
Particle diameter	μm	190
Granular viscosity	kg m^−1^ s^−1^	Gidaspow
Granular bulk viscosity	kg m^−1^ s^−1^	Lun et al.
Frictional viscosity	kg m^−1^s^−1^	Schaeffer
Frictional pressure	Pa	Based KTGF^a^
Frictional modulus	Pa	Derived
Granular temperature	m^2^ s^−2^	Algebraic
Solid pressure	Pa	Lun et al.
Radial distribution	–	Lun et al.
Elasticity modulus	Pa	Derived

*^a^Johnson and Jackson (for fixed regime)*.

#### Bubbling Regime

Once the air velocity exceeds a critical value (*U*_mf_), bubbles are generated above the air distributor and moves upwards tending to grow and coalesce. The pressure fluctuations across the bed are greatly influenced by the gas velocity because of the drag effect brought on the particles that ultimately reflects on the bubbles formation and motion (Qingcheng et al., 2011). To this purpose, a value of *U_o_* = 0.2 m s^−1^ (approximately 3.5 times the *U*_mf_) was used as boundary condition for the CFD simulations of the bubbling regime. In addition, a no slip condition was set both for the primary and the secondary phase at the wall. Given the primary importance of the drag effect in cold fluid dynamic applications and according to preliminary CFD tests and literature review (Esmaili and Mahinpey, 2011), the adjusted Syamlal–O’Brien model has been chosen and used for all CFD simulation in this work. More details about the solid phase properties and mathematical formulation that was set in the CFD model (for the bubbling regime) can be found in Table 4.

**Figure 5:**
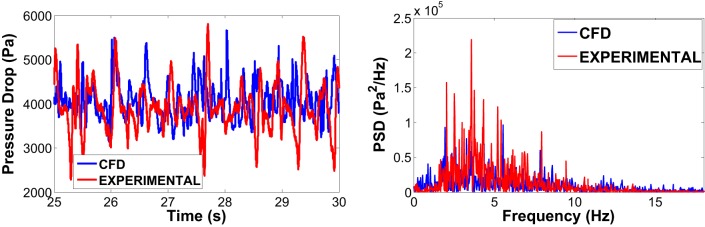
Experimental vs 2D two fluid model of a bubbling bed using alumina as fluidizing medium: comparison between the pressure drop signal in time (on the left) and its corresponding representation in the frequency spectrum (on the right).

Example of graphical outputs is shown in Figure 5 (comparison between experimental values and CFD simulations). Although there are similarities between the two set of data (Figure 5, left), the qualitative comparison of the pressure oscillation in time is not sufficient to assess the accuracy of CFD model in reproducing the experimental data. Figure 5 (right) shows a divergence between the PSD of the experimental and simulation signals (especially in the first part of the spectrum 0–2 Hz), which is mainly due to the intrinsic inability for the 2D model to capture and predict the exact “fingerprint” of bubbles. That might be due to the natural three dimensionality of the flow, supporting what found in previous works (Peirano et al., 2001; Acosta-Iborra et al., 2011).

In addition to the pressure drop signal, the distribution of the phase-volume fractions inside the bed is crucial and often used as a key validation point. However, the visual empirical observation of the stochastic evolution of flow patterns (bubble, cluster, channeling phenomenon, etc.) is rather challenging. Under the fluidization regime, bubbles move really fast and their presence close to the reactor wall is unpredictable. Their presence can be only observed in the wall proximity (in certain moments) and without any chance to evaluate what occurs deeper inside the system body. In addition, the presence of a thin layer of dust between bubbles approaching the reactor wall and the PVC wall itself further complicates the visual analysis.

### Model Sensitivity Analysis

The outputs of the 2D model sensitivity analysis are reported in the following along with the empirical data to also assess the accuracy of the numerical results. Together with the principle indicators of time-pressure drop, all the results were compared in terms of PSD cumulative (integral function) that summarizes at best the dynamic behavior of the bubbling system.

#### Restitution Coefficient

**Figure 6:**
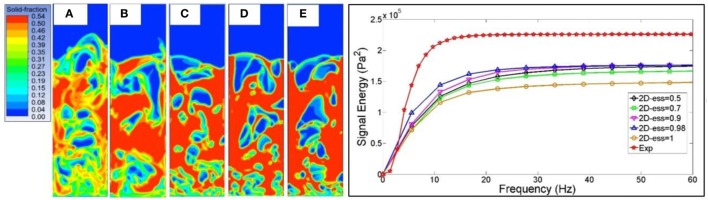
Left: solid volume distribution for *e_ss_* values of 1 **(A)**, 0.98 **(B)**, 0.9 **(C)**, 0.7 **(D)**, and 0.5 **(E)**. Right: corresponding PSD integral distribution.

As mentioned in Section “Hydrodynamic and Numerical Model,” this parameter quantifies the loss of energy due to the particles collisions, which impacts the momentum equation for the solid phase in Eqs 7, 8, 12, and 14. In this work, simulations were repeated using five different values of the restitution coefficient, in the 0.5–1 range, and results are compared in Table 5 and Figure 6.

**Table 5:** Comparison of main statistical indicators (of time-pressure drop) for the experiment and CFD simulations (varying the restitution coefficient—*e_ss_*).

*Δ*time 2–40 s	Time aver. *Δ*P (Pa)	Min. (Pa)	Max. (Pa)	Variance (Pa^2^)	Signal energy (Pa^2^) × 10^5^
2D—*e_ss_* = 0.5	4,049	2,395	6,669	178,208	1.765
2D—*e_ss_* = 0.7	4,052	2,738	6,163	167,538	1.679
2D—*e_ss_* = 0.9	4,051	2,310	7,625	175,998	1.762
2D—*e_ss_* = 0.98	4,082	2,694	5,934	176,553	1.764
2D—*e_ss_* = 1	4,102	2,922	6,434	150,331	1.490
Exp.	3,965	1,916	6,322	204,544	2.051

Results showed that the restitution coefficient does not have a significant impact on the CFD simulations outputs except when ideal collisions are assumed (*e_ss_* = 1). This is in agreement with the work of Tagliaferri et al. (2013) as well as with what was previously reported in open literature (McKeen and Pugsley, 2003; Zimmermann and Taghipour, 2005).

Esmaili and Mahinpey (2011) found comparable results concluding that when collisions becomes less ideal, particles become closely packed in the densest region of the bed resulting in sharper porosity contours and larger bubble. The simulation with *e_ss_* = 0.7 presented the best match with empirical data in terms of cumulated PSD, showing the lowest concentration of peaks in the first part of the spectrum as compared to the other simulations. In simulation where *e_ss_* = 1, the absence of sharp and big bubbles leads to a smaller variance of pressure drop and ultimately to a lower final signal energy (see Table 5 and Figure 6).

#### Solid Pressure

This parameter plays an important role in the momentum Eq. 2 for the granular phase and, along with shear stress tensor, contains all the parameters describing the intrinsic nature of granular flows.

Open literature shows no clear convergence on the best expression to be used for BFBs (Vejahati et al., 2009; Esmaili and Mahinpey, 2011) and, also for this reason, various formulations of the solid pressure term were investigated. Mathematical expressions for this term can be found in Section “Hydrodynamic and Numerical Model” according to Lun et al. (1984), Syamlal and O’Brien (1989), and Ahmadi and Ma (1990), respectively. This latter, differently from the first two, also embeds the frictional viscosity effects as shown in Eq. 16.

**Figure 7:**
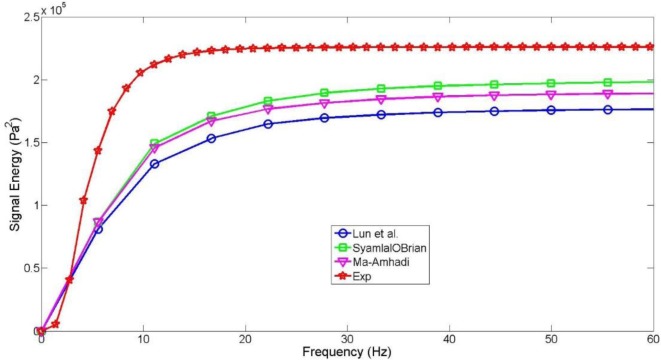
PSD cumulative trend for the experiment (red) and three CFD simulations based upon three different formulations of the solid pressure term.

Similarity between the Ma–Ahmadi and Syamlal–O’Brien model is depicted by the overlap within the 0–20 Hz range (Figure 7). The Syamlal–O’Brien model provides the best estimation in terms of the final total power achieved (with respect to the empirical data of our experimental bench, see Table 6). This model produced a slightly superior signal energy when compared to the Ma–Ahmadi expression. However, this little gap is due to the presence of peaks at frequencies higher than 20 Hz, which cannot be observed on the experimental PSD. This result may seem surprising since major contribution to particles momentum exchange arises from collisions in the dilute part of the bed and above all from particles friction, in the denser zones, which is accounted in the Ma–Ahmadi formulation through the frictional viscosity. However, in the TFM approach, the frictional viscosity is derived from the frictional pressure, which is only based on the solid fraction distribution inside the bed, and not on the real properties of solid particles such as their static, dynamic and rotational frictional components (that can be instead defined when using a discrete element method for particle–particle interactions). The importance of including a proper closure for particles friction, including also the rotational dynamic and effects (not accounted in this TFM study), has been very thoroughly explained and justified by Yang et al. (2016) in their recent TFM work.

**Table 6:** Comparison of main statistical indicators (of time-pressure drop) for the experiment and CFD simulations (varying the formulations for the solid pressure term—*P_s_*).

*Δ*time 2–40 s	Time aver. *Δ*P (Pa)	Min. (Pa)	Max (Pa)	Variance (Pa^2^)	Signal energy (Pa^2^) × 10^5^
2D—*P_s_* = Lun et al.	4,051	2,310	7,625	175,988	1.762
2D—*P_s_* = Syamlal–O’Brien	4,064	2,470	6,652	205,555	1.991
2D—*P_s_* = Ma–Ahmadi	4,077	2,729	6,935	189,681	1.899
Exp.	3,965	1,916	6,322	204,544	2.051

#### Minimum Fluidization Velocity (Drag Law)

In this study, focusing on a cold multiphase system, the drag force is the dominant term coupling the two phases. The adjusted Syamlal–O’Brien drag law was chosen because of his superior accuracy, also in agreement to what was previously found in literature (Vejahati et al., 2009; Min et al., 2010; Esmaili and Mahinpey, 2011). As explained in Section “Hydrodynamic and Numerical Model,” this drag law is particularly sensitive to the empirical value of the void fraction and the minimum fluidization velocity. While the former can be quite univocally computed (knowing the bed weight and the bed volume occupied by the solid phase when the fluidization onset occurs) the latter is often more complex to estimate (as we experienced in this case of study where the progressive transition between the fixed and the bubbling regime can be noticed). Three simulations corresponding to three different values of *U*_mf_ were carried out without modifying anything else in the operating condition setup or numerical settings.

**Figure 8:**
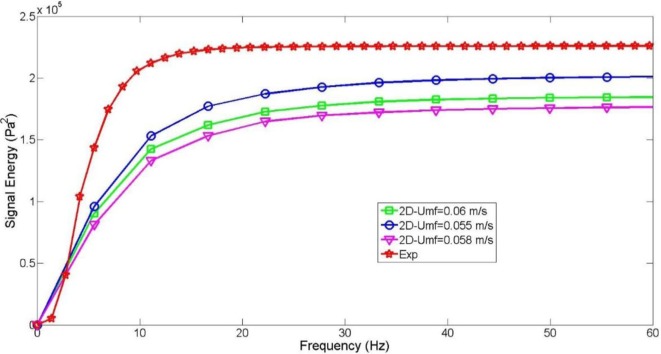
PSD cumulative trend for the experiment (red) and three CFD simulations based upon three different value of minimum fluidization velocity (*U*_mf_).

Results shown in Table 7 and Figure 8 depict the sensitivity of the model to a *U*_mf_ variation. The CFD simulation with *U*_mf_ = 0.055 m s^−1^ shows the best match with the empirical data in terms of pressure drop variance and final energy despite an overprediction of 500 Pa both for the maximum and minimum oscillation peaks found in the pressure drop signal. However, as already observed for the solid pressure analysis, the energy gap between the CFD simulation and empirical data has been reduced because of the frequency peaks over the 20 Hz, which are absent in the experiments. A better trend was found for the simulation with *U*_mf_ = 0.06 m s^−1^ with a minor growth of its PSD integral in the 20–60 Hz range (Figure 8). In this case (as also found for the other parameters investigated in this study), all the 2D model simulations showed some deficiency in reproducing the experimental PSD distribution with an unrealistic presence of peaks in the low frequencies zone. The model also depicted a weaker distribution of peaks in the 2–10 Hz range where the experimental PSD already reaches 90% of its total energy. Nevertheless, the mean pressure drop is correctly predicted in all of three cases, with a relative error found to be between 2 and 3% of the experimental one.

**Table 7:** Comparison of main statistical indicators (of time-pressure drop) for the experiment and CFD simulations (varying the *U*_mf_ used within the drag calibration algorithm).

*Δ*time 2–40 s	Time aver. *Δ*P (Pa)	Min. (Pa)	Max (Pa)	Variance (Pa^2^)	Signal energy (Pa^2^) × 10^5^
2D—*U*_mf_ = 0.06	4,051	2,393	7,637	184,297	1.853
2D—*U*_mf_ = 0.058	4,053	2,762	6,774	177,677	1.773
2D—*U*_mf_ = 0.055	4,082	2,557	7,146	207,083	2.021
Experimental	3,965	1,916	6,322	204,544	2.051

#### 2D vs 3D Models—Effects Induced by Numerical Geometry

This section focuses on the comparison of CFD results achieved by using 2D models (with two different mesh sizes corresponding to 10 and 20 times the particles diameter) and a 3D set with a relatively coarse grid (hexahedron of 3.81 mm side, namely, about 20 times the particles diameter) to restrain its computational costs. This particular comparison aimed at showing the potential improvements of 3D simulation while warranting its limitation in matching the total signal energy of the experiment as a result of coarse meshing. All the other numerical settings were the same for these simulations to have a fair comparison of the results.

**Figure 9:**
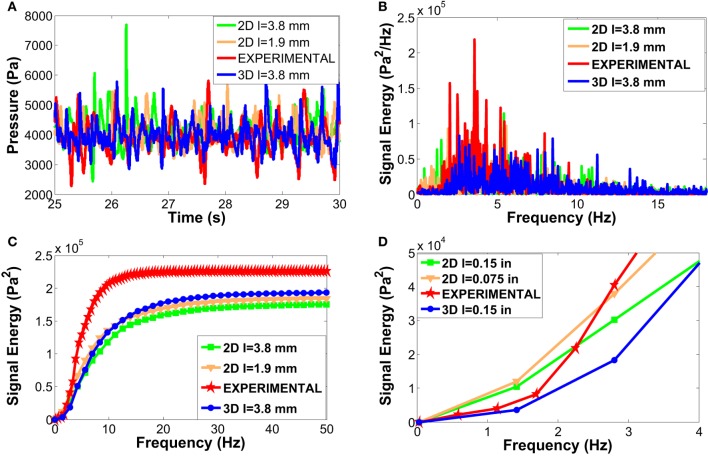
Comparison between the experiment and CFD of fluidized bed reactor: a time window of the pressure drop signals **(A)**, the corresponding PSD distributions **(B)**, the PSD integral curves in the range 0–50 Hz **(C)**, and its zoom in the range 0–4 Hz **(D)**.

Figures 9C,D show how the choice of a coarser mesh (3.81 mm in green vs 1.91 mm in orange) leads to an underestimation of the PSD distribution all over the frequency domain, which is particularly clear after the first 2 Hz. As for the grid sensitivity analysis [see Mesh Grid Sensitivity Analysis (2D Model)], the CFD model ability to capture the real distribution of bubbles as well as their sharp contours gradients is strongly linked to the mesh resolution (thanks to the reduce numerical diffusion). Consequently a finer grid allows for a better accuracy in the prediction of the pressure drop signal and its PSD distribution. Despite the overall general validity of this consideration, it is worth reminding that given the 3D nature of bubbles, the PSD should be used only as a qualitative tool in analyzing results coming from the 2D models. According to the present results for the 2D model (see Table 8), a coarser grid leads to an over prediction of the time-averaged pressure drop with a relative error of 4%, which is almost twice the error of the simulation with the finer grid.

**Table 8:** Comparison of main statistical indicators (time-pressure drop) for the experiment and CFD simulations (based upon two mesh grid size in 2D and using a full 3D geometry).

*Δ*time 2–40 s	Time aver. *Δ*P (Pa)	Min. (Pa)	Max (Pa)	Variance (Pa^2^)	Signal energy (Pa^2^) × 10^5^
2D—Δ = 3.81 mm	4,119	2,441	7,687	182,169	1.771
2D—Δ = 1.91 mm	4,051	2,393	7,637	184,297	1.853
3D—Δ = 3.81 mm	3,960	2,557	6,526	193,892	1.9465
Exp.	3,965	1,916	6,322	204,544	2.051

The PSD analysis of the 3D simulation (marked in blue in Figure 9) shows that the full geometry model does improve the match with the empirical data. This improvement emerges clearly from the observation of the first part of the PSD peaks distribution (0–2 Hz). Here, the 3D model and the experiment (marked in red) are in a very good agreement. This relevant improvement is also evident from the analysis of the PSD integral evolution in the 0–4 Hz range (see Figure 9D), where the divergence between 2D (green line) and 3D (blue line) simulations which were run with the same mesh grid, emerges clearly. However, slightly before 2 Hz, the 3D curve starts growing with a weaker intensity (as compared to the experiment) and this is most likely due to the coarse mesh used for this case, which was chosen to limit the duration of the 3D simulation. Further investigation will clarify and quantify the impact of the grid choice on the 3D model as it was done for the 2D case. Besides, the high frequency peaks (>15 Hz) are still present in the 3D simulation, which means that this error is independent of the 2D/3D approach and it might be an intrinsic limitation of the TFM approach. The presence of low frequency peaks was found to be a limitation of the 2D model, which could not be prevented by any parameters variation in the model sensitivity (performed in this work) and the extension to a full 3D model brought important improvement confirming what found and recommended by Peirano et al. (2001) and Acosta-Iborra et al. (2011) in their works.

### Physical Correlation between Pressure Drop and Void Fraction (Bubbles) Distribution

**Figure 10:**
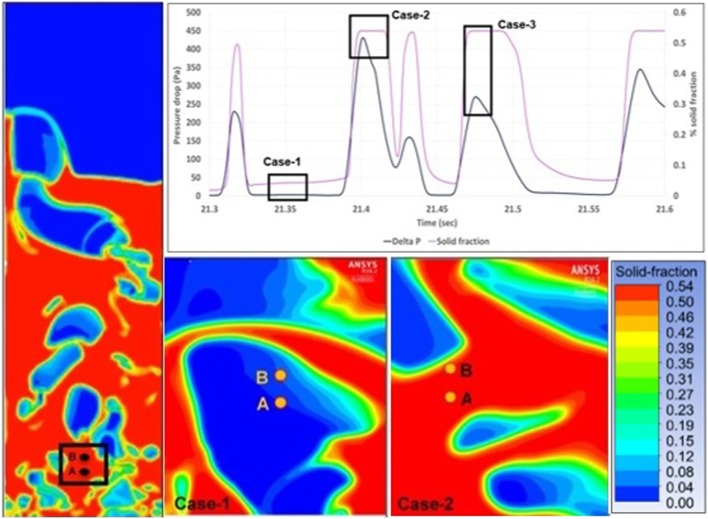
Correlation between bubbles distribution and pressure drop in bubbling bed reactor: a view of the whole bubble distribution as predicted by CFD-TFM along with the two points where pressure is monitored (on the left), the pressure drop trend vs solid fraction for a little time window (top), bubbles distribution in the area of the two points for different time (case-1 and case-2, bottom pictures).

The physical correlation between pressure drop and void fraction (bubbles) distribution it is quite complex due to the dampened effect of pressure waves propagating through the solid media. Specifically when a bubble reaches the surface the change in the voids distribution over the entire domain comes along with the generation of new pressure waves. However, there is always a certain delay in their propagation which result in a time lag of pressure variation. Because of this delay, along with the simultaneous bubbles eruptions and consequent changing of the voids distribution, it is difficult to correlate the pressure oscillations in time and the bubbles displacement. However, as shown by Acosta-Iborra et al. (2011), it is possible to simplify this analysis by considering the pressure difference between two points in the bed that very close to each other (see Figure 10, left hand side). This strategy allows correlating the local pressure drop with a single local bubble, rather than accounting for the global voids distribution in the whole bed. It is possible to locally apply the *Ergun* Equation, where the pressure drop is strictly linked to the void fraction and the gas velocity. Figure 10 shows two extreme cases, first (case-1), a single bubble embeds both check points. In such case the solid fraction drops to a value close to 0 because of the dearth of solid obstacles between point A and B. The lack of particles between the two points leads to an insignificant pressure drop. Case-2 shows the opposite situation, when both the check points are embedded in the emulsion phase (at high concentration of solid phase), which makes the fluid motion energetically expensive. In both cases the strong link between solid fraction and pressure drop is well depicted in the upper part of Figure 10. A third case is also possible, when the solid fraction is close to the maximum packing limit (like in case-2), but a lower value of pressure drop is predicted by the model. Such an occurrence is not surprising, since the gas velocity also plays a role in the gas pressure drop (as shown in the Ergun equation). According to the simulation, the gas velocity at the points A and B is 0.85 m s^−1^ for case-2 and 0.6 m s^−1^ for case-3, which explains the different simulated pressure drop.

## Conclusion

The results presented in this paper concern the application of a CFD TFM to a gas–solid fluidized bubbling bed reactor. The power spectral density (PSD) analysis (of pressure drop fluctuations) was used to compare the empirical data with the numerical predictions. The need of containing the computational costs was one of the priority and resulted in finding a flow time threshold for model simulations. Testing the effect of the sampling time on the empirical power spectral density (PSD) of pressure drop fluctuations it was found that 40 s could represent a good compromise to limit the duration of CFD simulations while ensuring the consistency of model validation with empirical data. The mesh size analysis carried out in this study showed that an interval spacing of 10 times the mean particle diameter was able to give acceptable results supporting what found in previous studies. Because of the unclear transition between fixed and bubbling regime, in the present experimental setup, the effect of *U*_mf_ (used as a parameter in the parametric drag law) on CFD simulations was investigated. The model outputs showed a better agreement with empirical data when the highest *U*_mf_ value (in the transition zone of the fluidization curve) was used. Beside in the ideal collision case (*e_ss_* = 1), the effect of the restitution coefficient appeared to be negligible on model predictions as well as the solid pressure term which was tested throughout two different formulations. In general, the 2D model revealed to correctly predict the time-averaged pressure drop and its fluctuations amplitude. Moreover, the post processing analysis of 2D simulations revealed a straightforward correlation between the pressure drop and void fraction distribution, confirming the presence of bubbles as the main source of local variation of pressure. A 3D version of the model was also implemented and compared with the 2D model. Despite being based on a “medium” size mesh, the 3D model drastically improved the results over the first part of the spectrum (0–2 Hz), namely, where all the previous 2D model simulations failed. The effect of a coarser grid on the numerical PSD was prior assessed allowing to believe how 3D model results may have been closer to the empirical ones also in the remaining part of the spectrum if a finer mesh was exploited. However, according to the simulation performances, reported in this work, this would result prohibitive from a computational standpoint especially in the perspective of a model scale up to industrial application. This barrier may possibly be overtaken if: (a) coarser particles can be used (which would result in a coarser mesh required to numerical verification); (b) a different type of variable analysis is needed possibly requiring a lower flow time as compared to the one used in this work; (c) the study involves macroscopic variables or type of analysis which do not require very fine mesh to be investigated.

## Notation

**Table:** 

*d_s_*	diameter of particles in the solid phase, m
*e_ss_*	restitution coefficient between colliding particles of solid phase
$\vec{\text{g}}$	vector representation of acceleration due to gravity, 9.81 m s^−2^
*g_o,ss_*	radial distribution function between particles of solid phase
*K_gs_*	momentum exchange coefficient between gas and solid phase, kg m^−3^ s^−1^
${\dot{\text{m}}}_{\text{pq}}$	mass flow rate from the generic phase *p* to the generic phase *q*, kg m^−3^ s^−1^
*P*	pressure, Pa
*P_s_*	solid pressure, Pa
*P*_frict_	frictional component of solid pressure, Pa
*t*	time, s
${\vec{\text{u}}}_{\text{q}}$	velocity vector of the generic (gas and solid) phase *q*, m s^−1^
${\vec{\text{u}}}_{\text{g}}$	velocity vector of gas phase, m s^−1^
${\vec{\text{u}}}_{\text{s}}$	velocity vector of solid phase, m s^−1^
${\vec{\text{u}}}_{\text{s}}{\;}^{\prime }$	velocity fluctuation vector of particles, m s^−1^

## Greek Letters

**Table:** 

α*_q_*	volume fraction of the generic (gas and solid) phase *q*
α*_g_*	volume fraction of the gas phase
α*_s_*	volume fraction of the solid phase
α_*s*,max_	maximum packing limit (volume fraction) of the solid phase
Θ*_s_*	granular temperature, m^2^ s^−2^
λ*_s_*	granular bulk viscosity, Pa s
μ*_g_*	viscosity of gas phase, Pa s
μ_*s*,tot_	total granular viscosity of solid phase, Pa s
μ_*s*,col_	collisional component of total granular viscosity, Pa s
μ_*s*,kin_	kinetic component of total granular viscosity, Pa s
μ_*s*,frict_	frictional component of total granular viscosity, Pa s
ρ*_q_*	density of the generic (gas and solid) phase *q*, kg m^−3^
ρ*_g_*	density of the gas phase, kg m^−3^
ρ*_s_*	density of the solid phase, kg m^−3^
${\bar{\bar{\tau }}}_{\text{g}}$	stress–strain tensor for the gas phase, Pa
${\bar{\bar{\tau }}}_{\text{s}}$	stress–strain tensor for the solid phase, Pa

## Author Contributions

LT made the experimental tests and part of the numerical simulation; he also was closely involved with the writing of the manuscript. TM helped supervise the different test both experimental and numerical simulations; he also contributed strongly to the manuscript redaction and reviewal. MB supervised the work helping to provide an industrial orientation; he was also involved in reviewing the manuscript. DC is co-PI of this work and codirector of LT; he was strongly involved in the reviewal of the manuscript. JL is the PI of this work; he was closely involved in the research orientation to start with; he supervised both LT and TM and contributed significantly to the manuscript reviewal.

## Conflict of Interest Statement

The authors declare that the research was conducted in the absence of any commercial or financial relationships that could be construed as a potential conflict of interest.
